# Single-cell dual-omics reveals translational and transcriptional landscapes and regulations in oocytes from ovarian endometriosis patients

**DOI:** 10.3389/fendo.2025.1534648

**Published:** 2025-02-17

**Authors:** Xiaoting Diao, Jiana Huang, Rui Xiang, Shaohong Zhuang, Qiqi Liang, Xiaoyan Liang, Haitao Zeng

**Affiliations:** ^1^ Reproductive Medicine Center, The Sixth Affiliated Hospital of Sun Yat-sen University, Guangzhou, China; ^2^ Guangdong Engineering Technology Research Center of Fertility Preservation, The Sixth Affiliated Hospital of Sun Yat-sen University, Guangzhou, China; ^3^ Biomedical Innovation Center, The Sixth Affiliated Hospital, Sun Yat-sen University, Guangzhou, China

**Keywords:** oocyte quality, ovarian endometriosis, oocyte meiosis, oxidative stress, RNA splicing

## Abstract

**Introduction:**

A significant proportion of women in their reproductive years are afflicted by endometriosis. And one of the major contributing factors to infertility linked to ovarian endometriosis is thought to be oocyte quality. The precise molecular mechanisms are still unknown. Furthermore, because of transcriptional silence, translatome is better able to explain molecular behavior in oocytes than transcriptome sequencing, which has been used widely in recent years.

**Methods:**

We conducted single-cell transcriptome and translatome sequencing on oocytes obtained from patients with ovarian endometriosis, as well as from control subjects with infertility due to tubal or male factors.

**Results:**

For the first time, we characterized the translational and transcriptional profiles of mRNA in GV-stage oocytes from patients with ovarian endometriosis and control subjects. Our translational analysis identified 2,480 differentially expressed genes in oocytes from ovarian endometriosis patients. Furthermore, we demonstrated that global translational activity in human oocytes is significantly altered by ovarian endometriosis. Key pathways such as "oxidative stress," "oocyte meiosis," and "spliceosome" were identified as critical factors influencing oocyte quality in ovarian endometriosis patients.

**Discussion:**

This study elucidated the molecular characteristics and potential mechanisms underlying poor oocyte quality in patients with ovarian endometriosis. Our findings provided new insights into the pathogenesis of endometriosis-associated infertility and highlighted potential therapeutic targets for improving oocyte quality and reproductive outcomes.

## 1 Introduction

Endometriosis (EMs) is characterized by the ectopic presence of endometrial tissue outside the uterus (1). Ovarian endometriosis (OE) is a common subtype of endometriosis (2). In recent years, many potential causes of OE-related infertility have been proposed, including gamete transport barrier, decreased ovarian reserve, impaired endometrial receptivity, immune dysfunction, and lowered oocyte and embryo quality (3–5). In particular, lower oocyte and embryo quality has a greater effect on adverse reproductive outcomes compared to impaired endometrial receptivity (6). Current research has shown that oxidative stress, mitochondrial dysfunction, morphological abnormalities, and arrested oocyte maturation can all have negative impacts on the quality of oocytes and embryos in patients with OE (7, 8). Nevertheless, deeper research into the underlying molecular mechanisms is required.

Previous study has conducted single-cell RNA sequencing on oocytes from patients with OE, which reveals the changes in steroid metabolism, oxidative stress, and cell growth regulation (9). Notably, when mammalian oocytes reach the germinal vesicle (GV) stage of the fully developed state, they cease to be transcriptionally active (10, 11). The maturation process of oocytes is almost totally dependent on post-transcriptional modifications and translational regulation of previous accumulating mRNA (10). Thus, rather than using the transcriptome to analyze molecular events in oocytes, the translatome is a more useful tool. Although great advances have been made in single-cell techniques, the majority of translatome strategies now in use require a significant number of cells (11, 12). For instance, hundreds of cells are still needed for ribosome profiling sequencing (Ribo-seq), which is currently more popular (11). Even low-input LiRibo-seq requires 100 to 250 oocytes/embryos and ultrasensitive Ribo-seq technique using 30-150 oocytes/embryos to detect the translational dynamics (11, 12). These methods are not feasible for precious human oocyte studies. Luckily, we have established a single-cell transcriptome and translatome sequencing(T&T-seq) method that can be used with a single oocyte (13, 14). Because human oocytes are rare, no translatome of human OE oocytes has been studied to yet. In this study, single-cell T&T-seq were used to map the dual-omics landscape of GV oocytes from OE patients. Here, we revealed the possible underlying mechanisms of the decreased oocyte competence and lower quality in human OE oocytes.

## 2 Results

### 2.1 T&T seq analysis of human OE and CON oocytes

**Figure 1:**
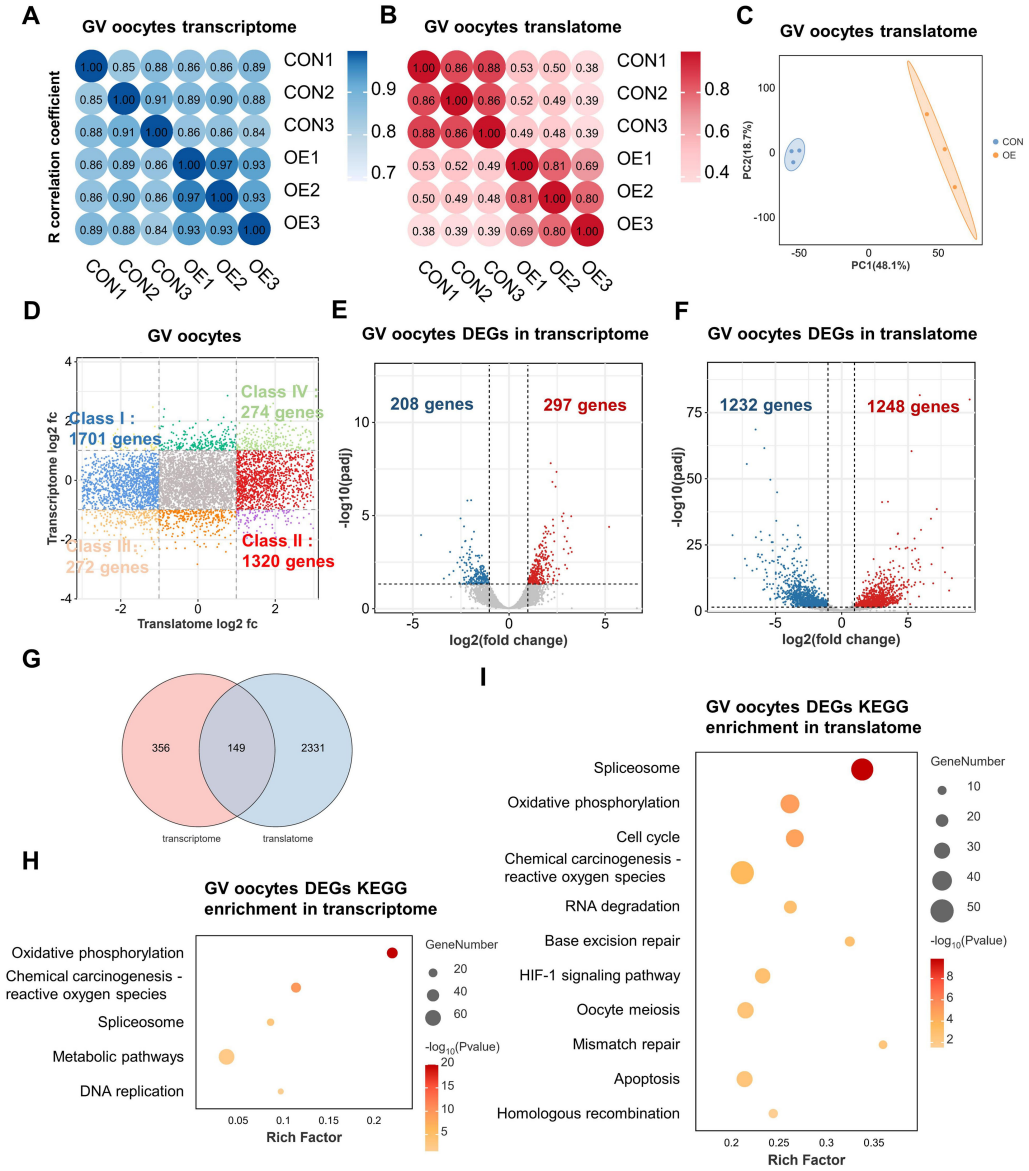
Single-cell translatome and transcriptome of OE and CON oocytes. **(A, B)** Correlation coefficient of the transcriptome **(A)** and translatome **(B)** data from OE and CON oocytes. **(C)** PCA plot of translatome sequencing data. Blue area covers CON oocytes and the orange area covers OE oocytes. **(D)** Scatter plot showing the difference in gene translation and transcription between OE and CON oocytes. Class I (blue) denotes genes translationally downregulated in OE oocytes but transcriptionally constant. Class II (red) denotes genes translationally upregulated in OE oocytes but transcriptionally constant. Class III (orange) denotes genes with downregulated translation and transcription in OE oocytes. Class IV (green) denotes genes with upregulated translation and transcription in OE oocytes. Downregulated, FC<0.5; upregulated, FC>2. **(E, F)** Volcano diagram showing DEGs detected by transcriptome **(E)** and translatome **(F)**. Red and blue dots denote up- and down-regulated genes, respectively. pagj < 0.05, FC>2 or <0.5. **(G)** Venn diagram showing the overlap of DEGs detected from the transcriptome (FC>2 or <0.5) and translatome(FC>2 or <0.5). **(H, I)** Representative KEGG analysis of DEGs detected by transcriptome **(H)** and translatome **(I)**. FC, fold change. DEGs, differentially expressed genes. KEGG, Kyoto Encyclopedia of Genes and Genomes.

OE-related infertility is closely associated with poor oocyte quality (15). To explore the underlying mechanisms of OE-related decreased oocyte competence, we next conducted single-cell T&T seq on human GV oocytes donated from the control-group (CON) and the OE-group patients. The Spearman correlation analysis revealed consistency and repeatability between samples between the two groups, each of which contained three biological duplicates (**Figures 1A, B**). Furthermore, principal component analysis (PCA) revealed that CON oocytes and OE oocytes clustered independently in translatomics and transcriptomics (**Figure 1C**, **Supplementary Figure S1**). In this study, the transcriptome of the CON and OE oocytes identified, respectively, 10708 and 11850 genes (transcripts per million (TPM) > 1). And in translatome, the oocytes from CON and OE identified 10,183 and 9926 genes (TPM > 1). The majority of OE oocyte-specific translationally repressed (class I, 1701 genes) or enriched (class II, 1320 genes) genes had constant transcriptional expression when we combined the transcriptome and translatome for analysis (**Figure 1D**). This finding is consistent with the widely accepted theory of transcriptional inactivation in fully developed oocytes (10). These results suggested that when examining the impact of OE on oocyte quality, the translatome is more significant than the transcriptome.

We next examined differentially expressed genes (DEGs) in OE and CON oocytes at the transcriptional and translational levels in order to obtain additional understanding of global differences in these two groups. In comparison to transcriptome, it was discovered that there were much more differences between the CON and OE groups in the translatome. The number of DEGs found in the translatome was substantially higher than that of the transcriptome, as seen by Venn and volcano plots (**Figures 1E-G**). That suggests a great deal of important information on oocyte gene expression will be missed by focusing solely on the transcriptome. Only 297 genes were found to be up-regulated and 208 genes were found to be down-regulated in transcriptome (**Figure 1E**). On the other hand, the translatome revealed that the expression of 1248 genes were upregulated and 1232 genes was downregulated in the OE group (**Figure 1F**). Remarkably, the overlap DEGs between the translatome and the transcriptome was limited to 149 genes (**Figure 1G**). These imply a lack of synchronization between transcription and translation in oocytes. We conducted an Kyoto Encyclopedia of Genes and Genomes (KEGG) enrichment analysis and discovered that the 149 genes have a correlation with “oxidative phosphorylation” and “reactive oxygen species” (**Supplementary Figure S2**). Moreover, the “oxidative phosphorylation,” “reactive oxygen species,” and “spliceosome” pathways were enriched in DEGs of both the transcriptome and the translatome in OE group oocytes, according to KEGG enrichment analysis (**Figures 1H, I**). However, translatome analysis offered more detailed pathway enrichment data and showed that the terms “cell cycle,” “RNA degradation,” “DNA repair,” “apoptosis” “progesterone-mediated oocyte maturation,” “homologous recombination,” and “oocyte meiosis” were linked to translational dysregulation in OE oocytes (**Figure 1I**). Hence, translatome, compared with the transcriptome, according to the central dogma, may be a more accurate indicator of oocyte quality and may offer further information regarding the lower oocyte quality observed in patients with OE. In addition, these results propose that oxidative phosphorylation, spliceosome, oocyte meiosis, apoptosis, RNA degradation, and DNA repair dysregulation may play key roles in OE-related decreased oocyte quality.

### 2.2 Downregulated DEGs analysis in translatome

**Figure 2:**
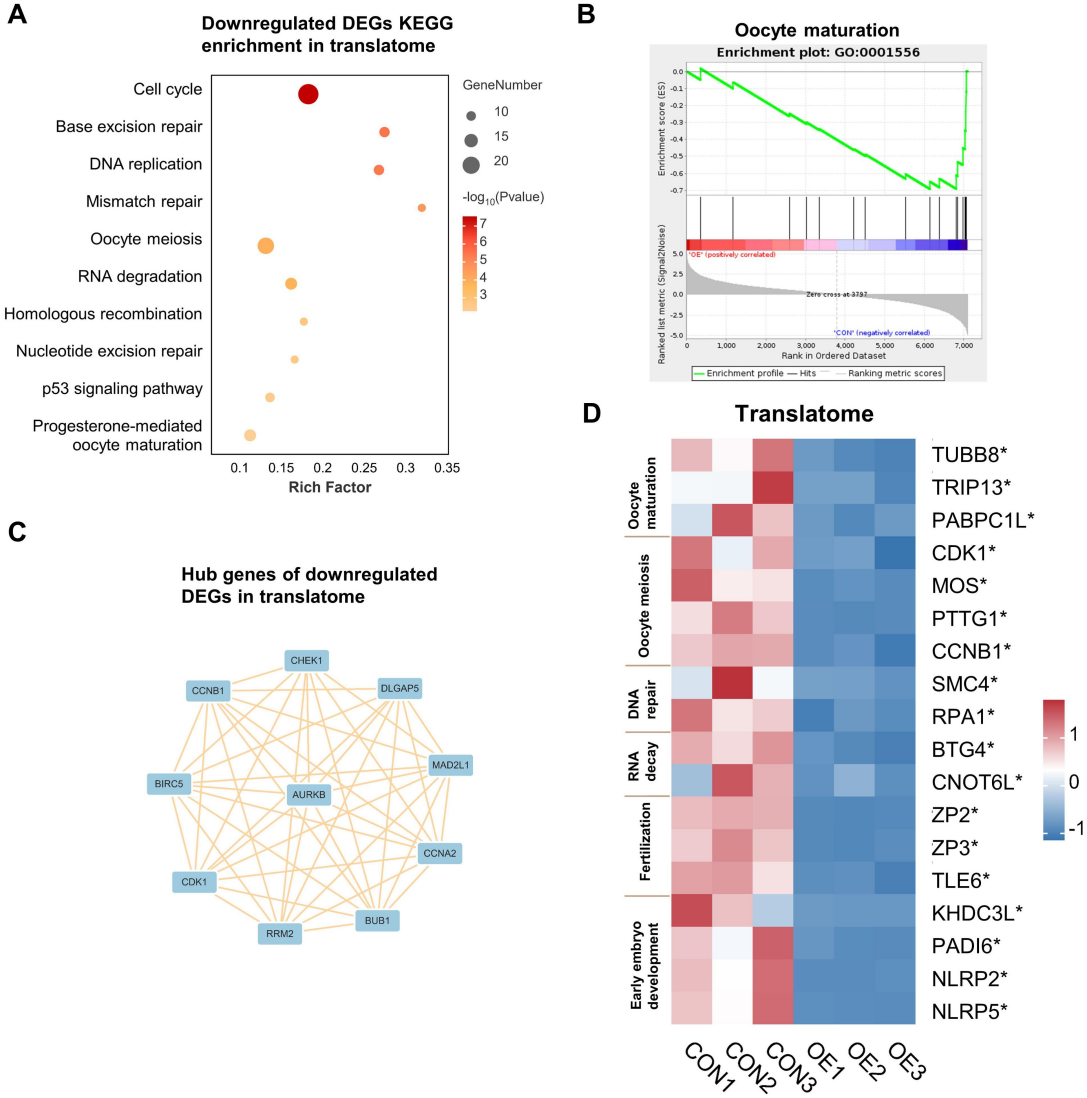
Downregulated DEGs analysis in translatome. **(A)** Representative KEGG analysis of downregulated DEGs detected by translatome. **(B)** Gene set enrichment analysis showing the translationally downregulated genes enriched in the oocyte maturation. **(C)** Cytoscape plots showing the hub genes in downregulated DEGs detected by translatome. **(D)** Translational expression heatmap showing specific genes in CON and OE oocytes. *p < 0.05. DEGs, differentially expressed genes. KEGG, Kyoto Encyclopedia of Genes and Genomes.

And then, the genes that were translationally up- and down-regulated in OE oocytes were analyzed independently. The down-regulated DEGs in the translatome were primarily enriched in terms of “cell cycle”, “DNA repair”, “DNA replication”, “RNA degradation”, “homologous recombination”, and “oocyte meiosis”, according to our KEGG analysis (**Figure 2A**). Gene Set Enrichment Analysis (GSEA) demonstrated that genes that are translationally down-regulated in OE oocytes are enriched in the oocyte maturation pathway (**Figure 2B**). Therefore, we propose that OE affects oocyte quality by reducing the translational expression of genes involved in the meiotic cell cycle and DNA damage repair in oocytes. Next, we conducted a Protein-Protein Interaction (PPI) analysis and filtered 10 hub genes according to degree values in order to gain a better understanding of the relationship among the down-regulated DEGs (**Figure 2C**). The main genes are CCNB1, CDK1, CHEK1, and AURKB, which are connected to oocyte meiosis (16–18).

In addition, we assessed the expression levels of genes linked to oocyte maturation, oocyte meiosis, DNA repair, RNA decay, fertilization, and early embryo development in oocytes (**Figure 2D**, **Supplementary Figure S3**). Surprisedly, we found a significant reduction in the expression levels of these genes, especially in the translatome. These discoveries advance our comprehension of the possible mechanisms responsible for the reduction in oocyte quality/capacity in OE.

### 2.3 Upregulated DEGs analysis in translatome

**Figure 3:**
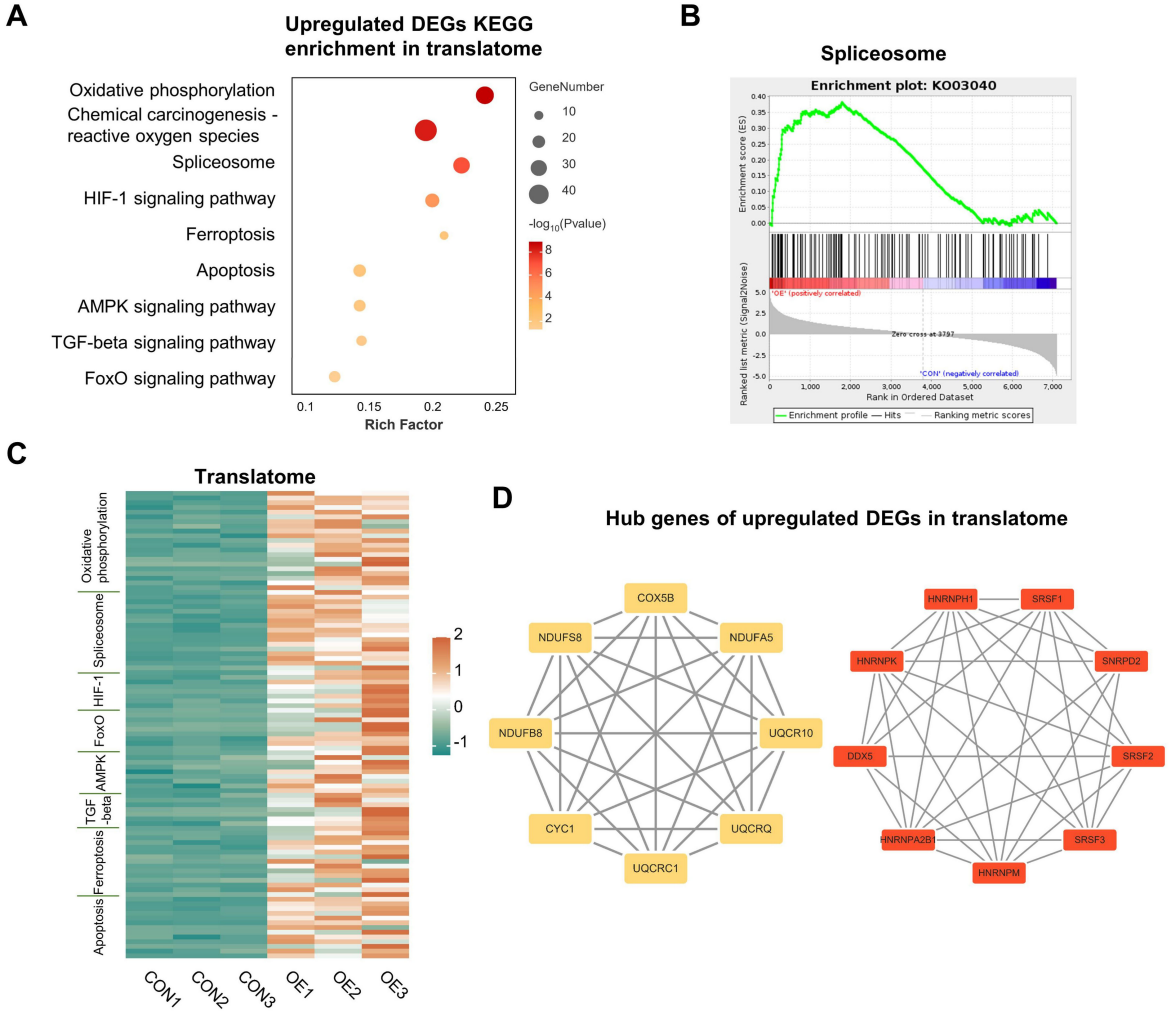
Upregulated DEGs analysis in translatome. **(A)** Representative KEGG analysis of upregulated DEGs detected by translatome. **(B)** Gene set enrichment analysis showing the translationally upregulated genes enriched in the spliceosome. **(C)** Translational expression heatmaps of genes in CON oocytes and OE oocytes with specific KEGG terms. **(D)** Cytoscape plots showing the hub genes in upregulated DEGs detected by translatome. DEGs, differentially expressed genes. KEGG, Kyoto Encyclopedia of Genes and Genomes.

In line with earlier research, we conducted KEGG analysis on the up-regulated genes in the translatome and discovered that they were primarily enriched in the pathways of “oxidative phosphorylation,” “reactive oxygen species,” “ferroptosis,” and “apoptosis”, which correspond with other research findings (**Figure 3A**) (19). Notably, the term “spliceosome” was also enriched (**Figure 3A**), which is further illustrated by the GSEA (**Figure 3B**). Previous studies have demonstrated a connection between oocyte DNA damage and RNA splicing (14, 20). But for the first time, we discovered that RNA splicing dysregulation might be a significant factor in the decline in oocyte quality brought on by OE. Each pathway-related gene’s translational expression in each sample is displayed on the heatmap (**Figure 3C**). We then carried out an analysis of PPI, and discovered that the top 30 hub genes were essentially split into two groups that were closely associated with RNA splicing and oxidative phosphorylation (**Figure 3D**). Hence, we discovered that, in addition to the previously documented elevated levels of oxidative stress, aberrant RNA splicing may contribute to the poor quality of OE oocytes.

### 2.4 Translational patterns in human OE oocytes

**Figure 4:**
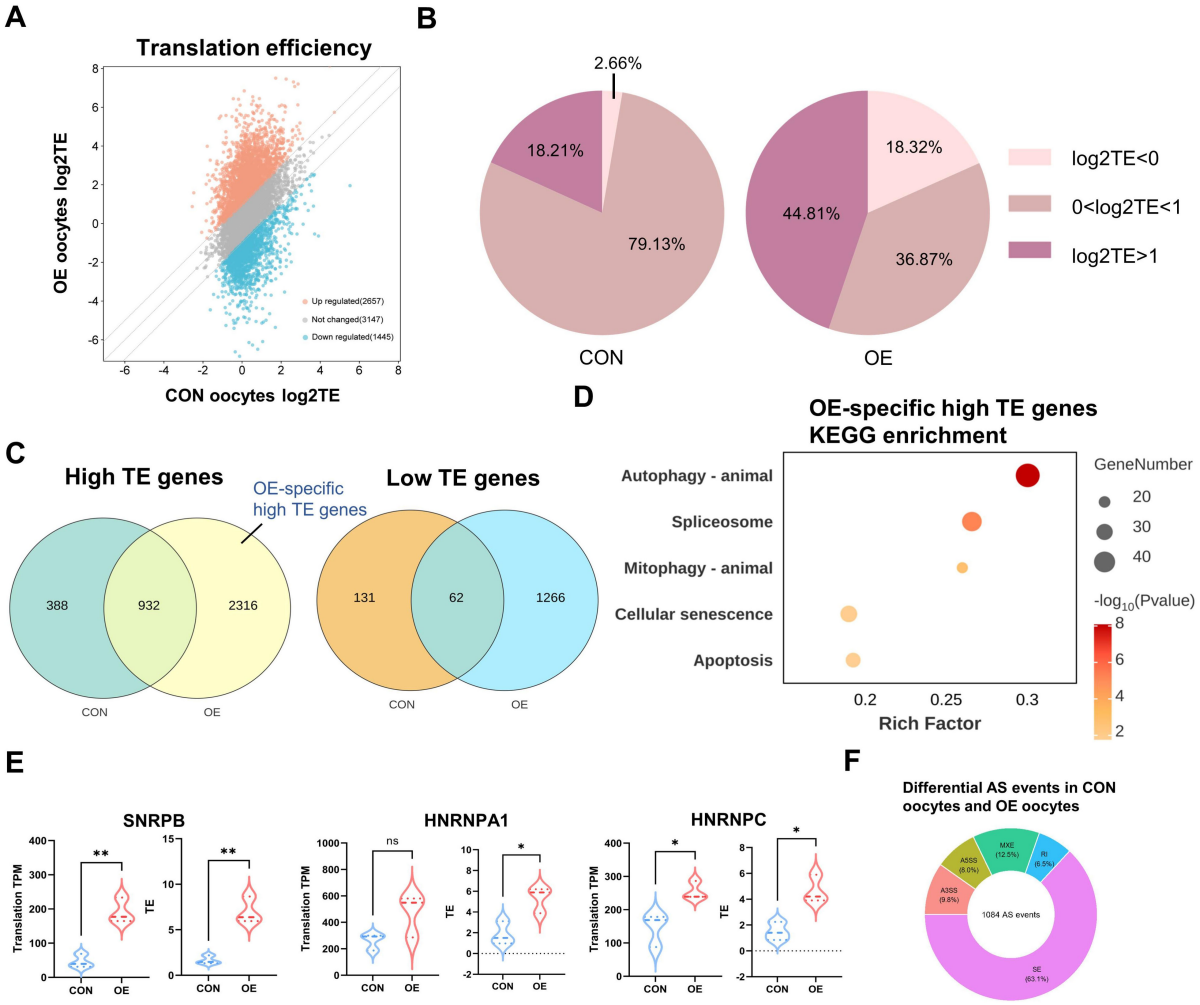
Distinct translatome pattern of CON and OE oocytes. **(A)** Scatter plot showing the RNA TE alterations of OE oocytes compared with CON oocytes. Orange and blue dots denote up- and down-regulated genes, respectively. Upregulated, FC>2; downregulated, FC<0.5. **(B)** Pie charts showing the proportion of TE in CON group and OE group respectively. **(C)** Venn diagram showing the overlap of low TE genes and high TE genes identified from CON and OE oocytes. **(D)** Representative KEGG analysis of OE high TE genes. **(E)** Translational expression levels of the representative RNA splicing-related genes in CON and OE oocytes. Data are shown as the mean ± SEMs. p-Values were calculated with Student’s t-test for independent samples. **(F)** Donut chart indicates the proportion of differential AS events in each category. AS, alternative splicing. SE, skipped exon, RI, retained intron, A5SS, alternative 5’ splice site, A3SS, alternative 3’ splice site, MXE, mutually exclusive exons. TE, translational efficiency. KEGG, Kyoto Encyclopedia of Genes and Genomes. ns, no significant difference. *p < 0.05, **p < 0.01, ****p < 0.0001.

Next, in order to gain a better understanding of the translation dynamics of OE oocytes, we determined the translation efficiency (TE) of highly expressed genes (TPM > 1 in transcriptome). When OE oocytes were compared to the controls, we discovered that large number of genes had abnormal translational suppression (1445 genes) and translational activation (2657 genes) (**Figure 4A**). The proportion of high TE genes (44.81%) and low TE genes (18.32%) in OE oocytes was significantly higher than those of CON (18.21% and 2.66%) (**Figure 4B**). Venn plots revealed that OE oocytes included 2316 high TE genes and 1266 distinct low TE genes (**Figure 4C**). This may indicate that OE oocytes have a modified global translational pattern. According to the DEGs of translational genomics, “spliceosome” was the KEGG-enriched terms for the OE-specific high TE genes (**Figure 4D**). Consistent with other research, this shows that abnormal RNA slicing are significant factors in the reduced quality of OE oocytes (14, 21, 22).

And then, we evaluated the translational efficiency of RNA splicing-related genes (**Figure 4E**), including SNRPB, HNRNPA1, and HNRNPC, which are crucial to the maternal to zygotic transition (MZT) and apoptosis pathway (20, 23, 24). In comparison to CON oocytes, their translation efficiency in OE oocytes was noticeably higher. Additionally, the phrases “autophagy” and “mitophagy” were similarly enriched in high TE genes (**Figure 4D**). These imply that one of the factors influencing oocyte quality may be the higher level of autophagy in OE oocytes. In summary, we have clarified the distinct translational pattern of OE oocytes and demonstrated that oocyte and embryo developmental potential may be impacted by abnormal activation of high TE genes related to RNA splicing and autophagy. To explore the RNA splicing events in OE group oocytes in more detail. We used replicate multivariate analysis of transcript splicing (rMATS) to examine differential splicing events in the CON and OE transcriptome. Skipped exon (SE), retained intron (RI), alternative 5’ splice site (A5SS), alternative 3’ splice site (A3SS), and mutually exclusive exons (MXE) were among the 1084 differential splicing events found in the OE group as compared to the CON group. SE was the most common differential splicing event (63.1%) (**Figure 4F**). According to published research, the abnormalities in the alternative splicing (AS) of key factors have been associated to translational efficiency of their target genes (25).

## 3 Discussion

It has been widely reported that female fertility may be negatively impacted by OE (26), presenting decreased number of retrieved oocytes, number of available embryos, top-quality embryos rate, blastocyst formation rate, and cumulative live birth rate (27, 28). Hence, there is an urgent need to elucidate the underlying mechanisms of OE-associated declined oocyte quality and competence. As fully grown GV-stage oocytes are in transcriptional arrest, the post-transcriptional modifications and translational regulation of mRNAs stored in advance is a crucial step in oocyte maturation and oocyte-to-embryo transition (OET) (10). Up to now, owing to the cell number requirement of translatomics and limitations of human oocytes not much is known about the mRNA translational landscapes in OE oocytes. Here, we first conducted single cell T&T-seq to document the translation and transcriptional patterns of mRNA in GV-stage oocytes donated from the OE and CON patients. The alterations in gene expression between OE and CON oocytes were investigated. Single cell T&T seq revealed the global translational activity of human oocytes was affected by OE. More importantly, the translationally altered genes were transcriptionally constant, which is in consensus with the classical theory that fully grown oocytes are transcriptional silence (29). Besides, it highlights the value of this study that, compared with transcriptomics, translatomics can provide deeper information of how OE affect oocyte quality and developmental competence. “Oxidative stress”, “oocyte meiosis”, and “spliceosome” were identified as the critical pathway that affected OE-oocyte quality, which may be the potential targets for improve oocyte quality.

Noticeably, the down-regulated DEGs in the translatome were primarily enriched in terms of “cell cycle”, “DNA repair”, “DNA replication”, “RNA degradation”, “homologous recombination”, and “oocyte meiosis”. Repairing the damage later in the cell cycle, as rather as during the resumption of meiosis, has selective advantage (30). Inherited errors related to DNA repair may result from upregulation of the DNA repair pathway in OE oocytes at the GV stage (30). In addition, dysregulation of maternal mRNA degradation leads to oocyte dysmaturity and embryo arrest (31). Downregulation of BTG4 and CNOT6L, key factors in RNA degradation also observed in this study (31). Furthermore, it has been reported that recurrent meiotic anomalies in oocytes *in vitro* maturation (IVM) from EMs patients are correlated with a possible meiotic phase I delay or impairment (32). Additionally, ferroptosis in oocytes and cumulus cells is caused on by iron-overloaded follicular fluid, which results in oocyte dysmaturity (33, 34). And hub gene analysis of translational inhibited genes in our study further showing the main genes are CCNB1, CDK1, CHEK1, and AURKB, which are connected to oocyte meiosis (16–18). Noticeably, CCNB1 and CDK1 play key roles in oocyte GVBD promotion (18). CHEK1 is one of the G2/M checkpoints, involving in DNA replication during the S-phase of cell division (17). And the quality of oocytes and embryos was favorably linked with BIRC5 protein levels (35). In addition, down-regulation of other genes, detected by translatome, may also affect the quality of OE oocytes. For example, TUBB8 is involved in human oocyte spindle assembly, and mutations in this gene cause oocyte developmental arrest at the MI stage (36). Moreover, PADI6, NLRP2, and NLRP5 are components of the subcortical maternal complex (SCMC), which is essential in embryo activation (36, 37). Therefore, cell cycle dysregulation is important in OE-related declined oocyte quality, which may help provide future therapeutic targets to improve the quality of OE oocytes.

On the other hand, our research indicated that the translationally altered genes enriched in the “oxidative phosphorylation”, “reactive oxygen species”, “DNA repair”, “cell cycle”, “progesterone-mediated oocyte maturation,” “homologous recombination,” and “oocyte meiosis” pathways. Previous studies have shown increased oxidative stress damage in OE patients’ ovarian cortex, follicular fluid, and granulosa cells (19). Reactive oxygen species (ROS) may cause meiotic arrest through inducing DNA damage and disturbing the spindle assembly checkpoint (38). Further evidence shows that endometriosis causes cell cycle dysregulation then affecting oocyte quality. For example, mouse oocytes collected from either endometriosis models or exposed to human serum, peritoneal fluid and follicular fluid from patients with endometriosis show spindle abnormalities and incomplete extrusion or division of the first polar body, which are associated with decreased oocyte quality and early embryo development rate (38–41). Furthermore, translatome analysis of up-regulated genes also pointed to aberrant regulation of the pathways for reactive oxygen species and oxidative phosphorylation. Strikingly, hub genes analysis screened CYC1, COX5B, NDUFB8, and UQCRQ, which are all parts of complexes in the respiratory electron transport chain of the mitochondria (42–45). The aberrant translations of these genes may cause mitochondrial dysfunction, leading to decrease the capacity of inhibiting ROS creation (46). The above results and studies emphasize the critical role of oxidative stress in OE-induced decreased oocyte quality and developmental competence. Hence, antioxidants are recommended as therapeutic options to improve oocyte quality in patients with endometriosis (41, 47, 48).

Interestingly, single cell T&T seq in this study offers us a fresh perspective that RNA splicing may be critically involved in the molecular mechanisms underlying OE-related declined oocyte quality. Noticeably, AS is a crucial post-transcriptional mRNA processing mechanism in GV oocytes, the abnormalities of which may adversely impact oocyte and embryo development (14, 49). For instance, RNA splicing has been reported that it is important for preserving the integrity of the oocyte transcriptome and for oocyte maturation (21, 22). Moreover, it has been demonstrated that splicing errors at the ZGA are developmentally planned and evolutionarily conserved in mammalian embryos, potentially attenuating the cellular response to DNA damage (20). On the other hand, an enhanced DNA damage response caused by the overexpression of SNRPB, a spliceosome component, before the ZGA stage may affect early embryo development (20, 50). SE events are impacted by SNRPB, and our results also indicate that OE oocytes present aberrant SE events (20). Furthermore, in zebrafish, overexpression of the splicing factor HNRNPA1 lengthens the maternal mRNA’s poly(A) tail and boosts translation efficiency, which could account for the significantly higher proportion of high-TE genes in OE oocytes compared to CON oocytes (23). And HNRNPC over-expression encourages apoptosis (24). Hence, these suggest RNA splicing dysregulation may play an essential role in the decreased oocyte quality and developmental competence in OE patients. However, further research should be undertaken to prove the hypothesis.

This study has a few limitations. We only had three biological replicates per group since human oocytes were scarce. Individual variations may still have an impact on the outcomes of data analysis despite this. Furthermore, we didn’t perform out any functional verification examinations. We therefore intend to look more closely at the detailed biological pathways through which aberrant RNA splicing impacts the quality of OE oocytes. Moreover, related therapies are looked for to help OE patients have better reproductive outcomes.

In summary, we first used T&T-seq to map the transcription and translation of OE oocytes. The findings suggest that the translation pattern of OE oocytes is distinct. Our findings include the identification of numerous new genes linked to oxidative stress damage and meiosis, which will aid in the exploration of the fundamental mechanisms behind the OE oocyte quality reduction and the identification of potential treatment targets. Furthermore, it was first proposed that OE oocytes had dysregulated RNA splicing. This offers a fresh viewpoint for researching the molecular processes by which OE impacts oocytes.

## 4 Methods

### 4.1 Human GV oocytes collection

Women identified with male factor infertility or tubal infertility who underwent intracytoplasmic sperm injection (ICSI) between July and August 2024 were included in control (CON) group. Women who underwent ovarian stimulation and had endometriomas identified via transvaginal ultrasound were categorized into the ovarian endometriosis (OE) group. Ovarian stimulation was induced using a short protocol of gonadotropin-releasing hormone (GnRH) agonist. Transvaginal sonography was employed to monitor follicular maturation, and a 250 μg injection of human chorionic gonadotropin (HCG) was administered to trigger ovulation when the dominant follicles measured 18 mm in diameter. Transvaginal needle-guided oocyte extraction was executed, followed by enzymatic treatment with hyaluronidase and gentle pipetting removal of cumulus cells. Germinal vesicle (GV) oocytes were rinsed twice with phosphate-buffered saline (PBS) and collected for single-cell transcriptome and translatome sequencing (T&T-seq). Three GV oocytes from both OE and control groups were analyzed using T&T-seq. This collection was performed with the consent of the donors and was approved by the Ethics Committee of The Sixth Affiliated Hospital of Sun Yat-sen University, in compliance with the relevant certification(2024ZSLYFEC-001).

### 4.2 Transcriptome and translatome sequencing

The procedure was executed based on established protocols. Oocytes were lysed in a mixture of lysis buffer and RNase inhibitor (Vazyme, N712) on ice for 20 minutes. The lysates were split into two portions; one portion was for transcriptome, and the other was for translatome. For the latter, RiboLace beads (Immagina, RL001) were prepared as per the manufacturer’s instructions and each sample was mixed with the beads in a binding buffer containing various components. This mixture was incubated at 4°C for 1 hour. The beads were then washed using the W-buffer (Immagina, RL001) on a magnetic stand. Subsequently, they were resuspended in a solution containing RLT buffer (Qiagen,74 004) and 10% of beta-mercaptoethanol and 1% of glycoblue, followed by a short incubation at room temperature. The supernatant was moved to a new PCR tube, leaving the beads. The ribosome-bound full-length RNA was isolated using LiCl and VAHTS RNA Clean Beads (Vazyme, N412), following the manufacturer’s protocol. Both the total RNA and ribosome-bound RNA were reverse transcribed to synthesize cDNA, which was amplified for 20 PCR cycles. The cDNA was purified and quantified using the protocol of Single Cell Full Length mRNA-Amplification Kit (Vazyme, N712). VAHTS DNA Clean Beads (Vazyme, N411) were used to purify the cDNA amplification products. And the integrity was confirmed using a Bioanalyzer 2100, showing a peak at approximately 2000 bp. The construction of indexed libraries was accomplished using the TruePrep DNA Library Prep Kit V2 (Vazyme, TD502), which is designed for Illumina sequencing platforms. The VAHTS DNA Clean Beads (Vazyme, N411) were employed to selectively isolate and purify the amplified DNA fragments, targeting a size range of 250–450 bp. The libraries were quantified and quality-checked using Qubit and Bioanalyzer 5400. Pair-end sequencing was conducted on an Illumina Novaseq XP platform using a PE150 mode.

### 4.3 T&T-seq data analysis

The raw reads were quality-trimmed using Trim Galore, and the cleaned reads were aligned to the human genome (hg38) with Hisat2. The gene annotation files were obtained from the Gencode database and GenBank. Read counts were calculated using Featurecounts, and transcript abundance was determined in TPM. DEGs (Pagj < 0.05, Fold Change>2 or <0.5) were identified using DESeq2, focusing on genes with a TPM>1. The translational efficiency (TE) was calculated by dividing the the adjusted TPM values (TPM + 1) of the translatome by that of the transcriptome. TPM and TE were compared between groups using the student’s t test. P < 0.05 was considered significant. rMATS was used to analyze the splicing events. Events with Δpercent-spliced-in (PSI)>|0.15| and false discovery rate (FDR) < 0.05 were regarded as differential splicing events.

### 4.4 Spearman correlation analysis

Spearman correlation analysis was conducted utilizing the GENE DENOVO web-based tool. The analysis parameters were set to their default values.

### 4.5 Principle component analysis and functional enrichment analysis

Principle Component Analysis, Kyoto Encyclopedia of Genes and Genomes (KEGG) and Gene Set Enrichment Analysis (GSEA) were conducted utilizing the GENE DENOVO web-based tool. The analysis parameters were set to their default values. Statistical significance was determined at the P < 0.05 threshold.

### 4.5 Protein-protein interaction and hub genes analysis

The Search Tool for the Retrieval of Interacting Genes (STRING) database built the PPI network. After determining degree values, hub genes were filtered using the Cytohubba plugin for Cytoscape (3.10.0).

## Data Availability

The datasets presented in this study can be found in online repositories. The names of the repository/repositories and accession number(s) can be found below: https://ngdc.cncb.ac.cn/gsa-human, HRA008738.
