# Supplementary material for: Single-cell dual-omics reveals translational and transcriptional landscapes and regulations in oocytes from ovarian endometriosis patients
Source: Front Endocrinol (Lausanne). 2025 Feb 17;16:1534648. doi: 10.3389/fendo.2025.1534648 (PMC11872718; doi:10.3389/fendo.2025.1534648)
Supplement: Supplementary file 1 [file DataSheet1.docx]

Supplementary Material

# Supplementary Figures


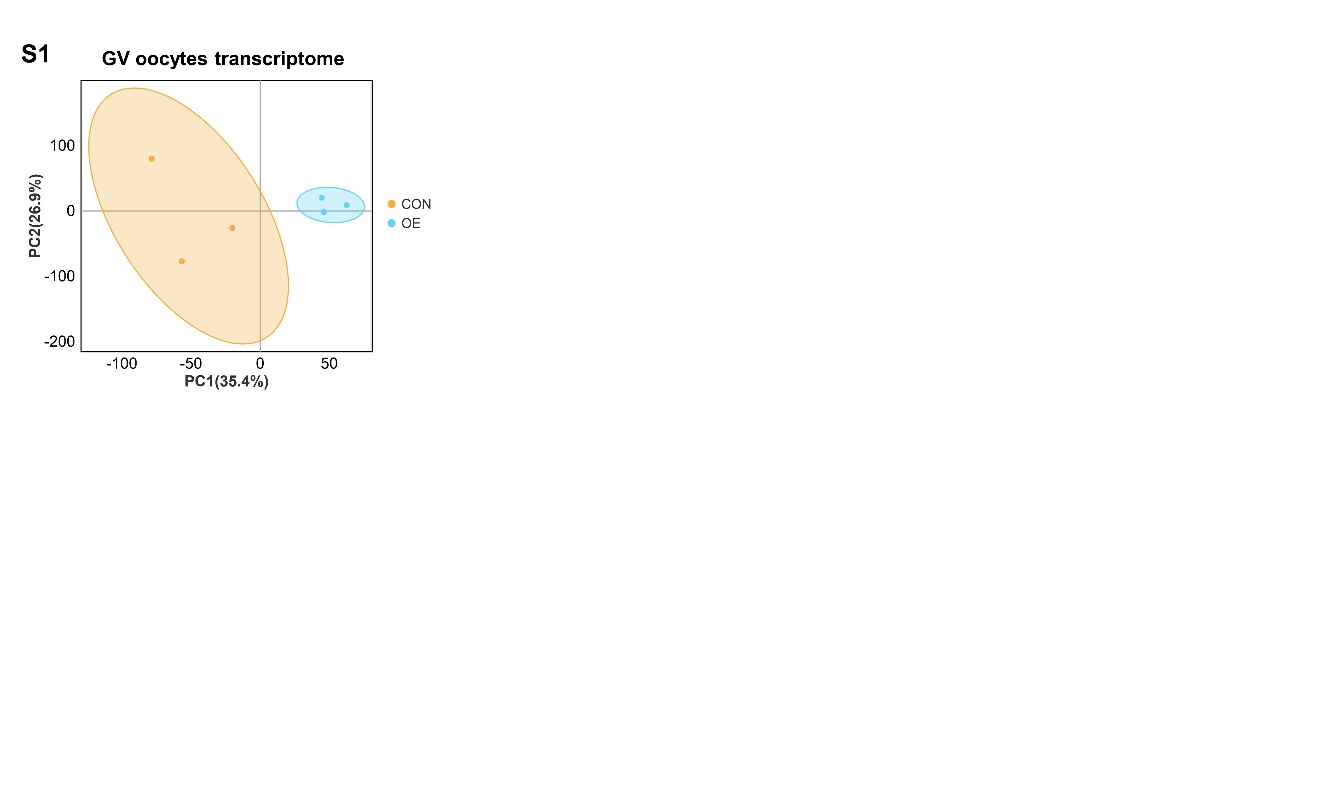
**Supplementary Figure 1.** PCA plot of transcriptome sequencing data. Orange area covers CON oocytes and the blue area covers OE oocytes.


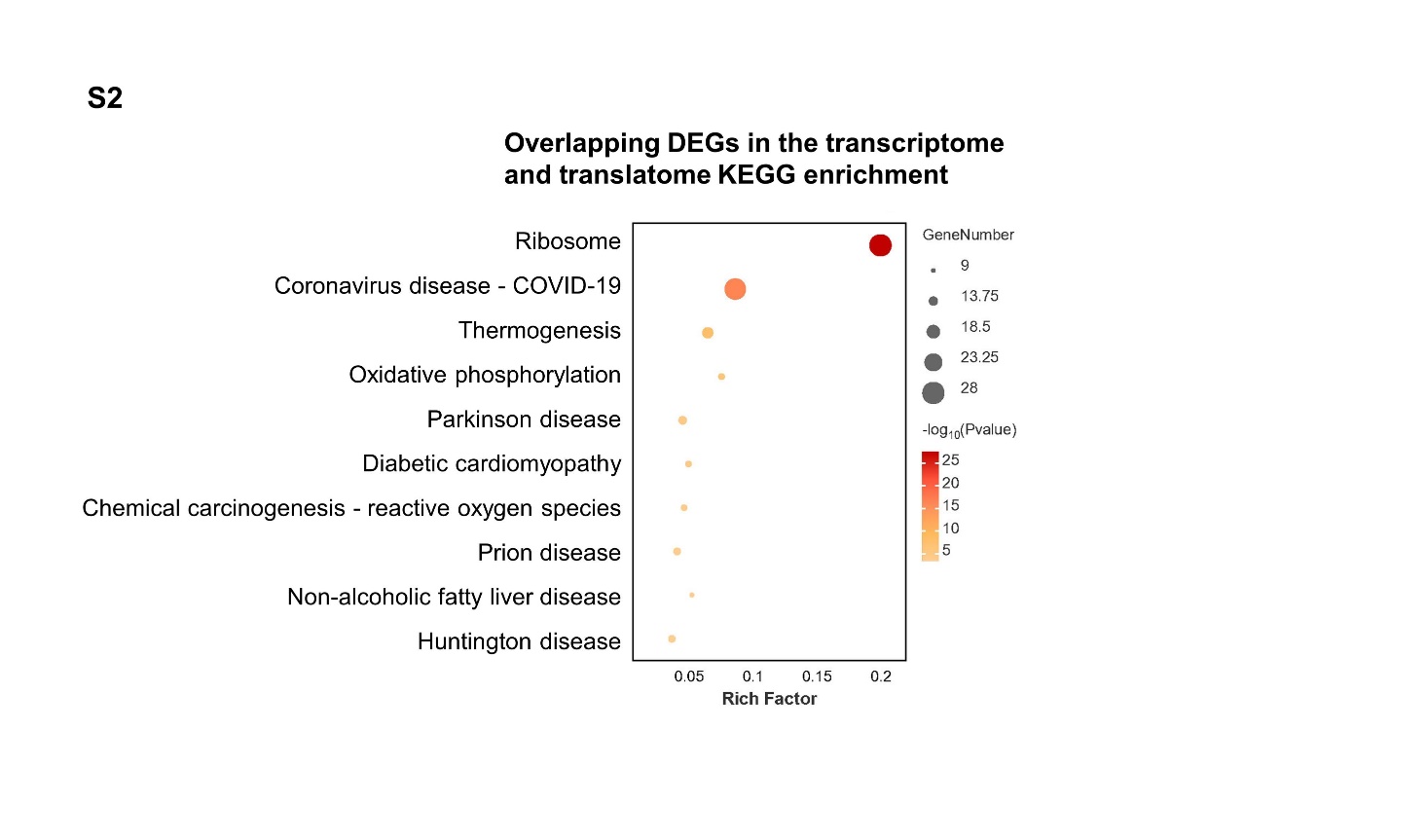


**Supplementary Figure 2.** Top 10 KEGG enrichments of overlapping DEGs in the transcriptome and translatome.

**
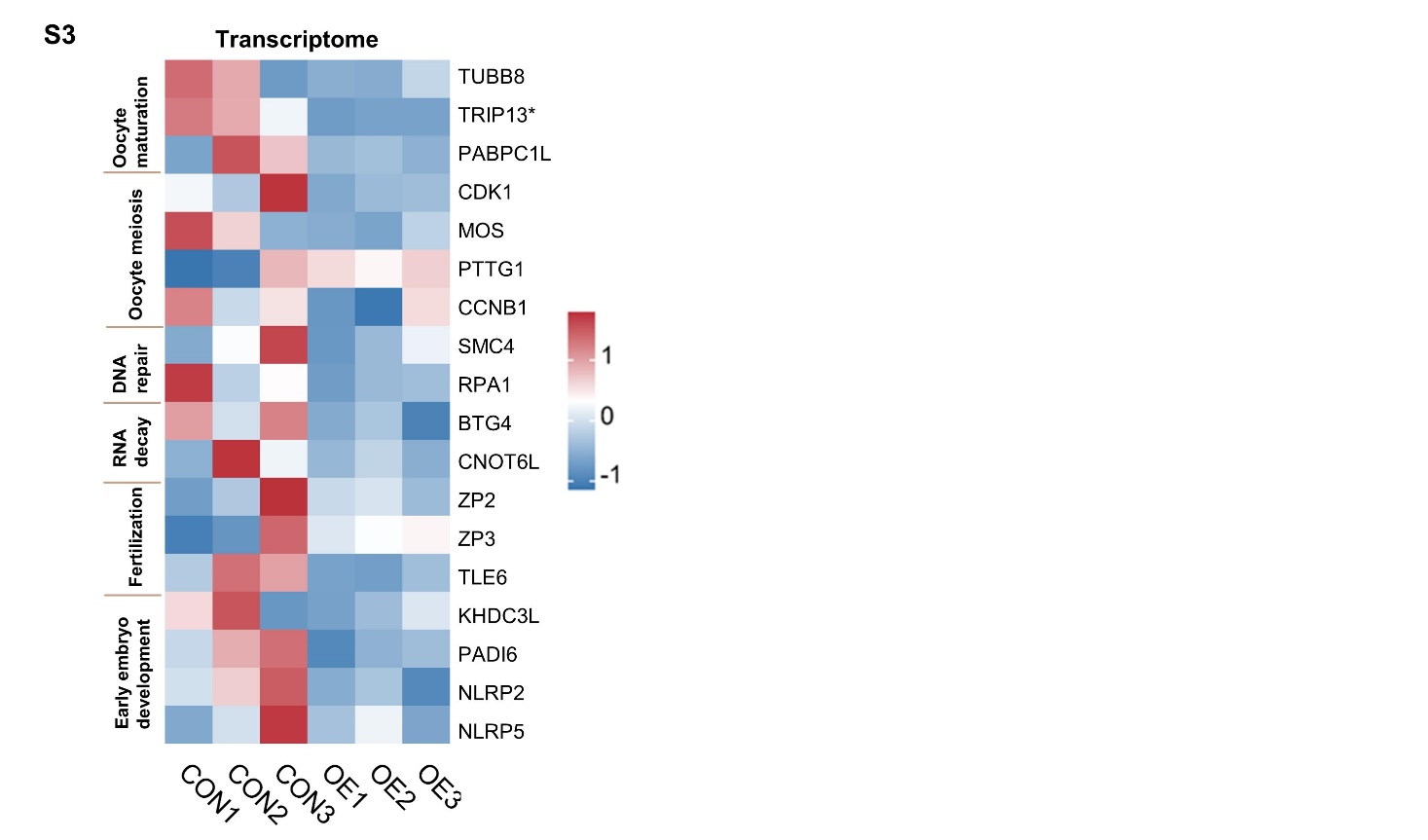
**

**Supplementary Figure 3.** Transcriptional expression heatmap showing specific genes in CON and OE oocytes. *p < 0.05.
